# Short Sequence Aligner Benchmarking for Chromatin Research

**DOI:** 10.3390/ijms241814074

**Published:** 2023-09-14

**Authors:** John Lawrence Carter, Harlan Stevens, Perry G. Ridge, Steven Michael Johnson

**Affiliations:** 1 Department of Microbiology and Molecular Biology, Brigham Young University, Provo, UT 84602, USA; jlawcar@gmail.com (J.L.C.); harlanstevens144@gmail.com (H.S.); 2 Department of Biology, Brigham Young University, Provo, UT 84602, USA; perry.ridge@byu.edu; 3 Neuroscience Center, College of Life Sciences, Brigham Young University, Provo, UT 84602, USA

**Keywords:** alignment programs, ChIP-seq, NGS

## Abstract

Much of today’s molecular science revolves around next-generation sequencing. Frequently, the first step in analyzing such data is aligning sequencing reads to a reference genome. This step is often taken for granted, but any analysis downstream of the alignment will be affected by the aligner’s ability to correctly map sequences. In most cases, for research into chromatin structure and nucleosome positioning, ATAC-seq, ChIP-seq, and MNase-seq experiments use short read lengths. How well aligners manage these reads is critical. Most aligner programs will output mapped reads and unmapped reads. However, from a biological point of view, reads will fall into one of three categories: correctly mapped, incorrectly mapped, and unmapped. While increased sequencing depth can often compensate for unmapped reads, incorrectly and correctly mapped reads appear algorithmically identical but can produce biologically significant alterations in the results. For this reason, we are benchmarking various alignment programs to determine their propensity to incorrectly map short reads. As short-read alignment is an important step in ATAC-seq, ChIP-seq, and MNase-seq experiments, caution should be taken in mapping reads to ensure that the most accurate conclusions can be made from the data generated. Our analysis is intended to help investigators new to the field pick the alignment program best suited for their experimental conditions. In general, the aligners we tested performed well. BWA, Bowtie2, and Chromap were all exceptionally accurate, and we recommend using them. Furthermore, we show that longer read lengths do in fact lead to more accurate mappings.

## 1. Introduction

In today’s world of next generation sequencing (NGS) and high-throughput biology, sequence read mapping has become, at most, an afterthought. With the ease of web-based graphical user interfaces like Galaxy [1], any biologist can easily undertake bioinformatic tasks, like sequence read mapping, which were previously left to the experts. While this is overwhelmingly a positive development, it can also be a cause for concern. Mistakes made at the sequence read-mapping step of the data processing pipeline can have drastic downstream effects. Galaxy itself issues a warning for short-read alignment programs it supports: “There is no such thing (yet) as an automated gearshift in short read mapping. It is all like stick-shift driving in San Francisco. In other words, running this tool with default parameters will probably not give you meaningful results. A way to deal with this is to understand the parameters by carefully reading the documentation and experimenting. Fortunately, Galaxy makes experimenting easy” [1].

The intent of this study is to provide useful information so that those with limited bioinformatic experience who are undertaking short-sequence high-throughput assays, such as ATAC-seq, ChIP-seq, and MNase-seq, can make informed decisions about alignment programs. To this end, we use two different approaches, one using biological data and the other using in silico derived simulated reads. In the first approach, we cross-compare several aligner programs’ performance using real biological ChIP-seq data to see to what extent ChIP-seq results will differ based solely on the aligner used. With the second approach, we generate simulated sequence reads to quantify the accuracy of each aligner programs’ alignments.

As noted above, there are two main ways to evaluate alignment programs, using simulated data sets or biological data sets. The benefit of using real data (biological) is that it is derived from real biological samples and conditions. The downside with testing biological data is that your output only tells you if reads mapped or did not map to the genome. There is no way of knowing if the reads are correctly mapped to the loci from which they were derived. Most benchmarking studies have used real data [2]. Conversely, using simulated data allows us to know if the reads are correctly mapped or not to the loci of origin. The downside of simulated data is that it is hard to exactly replicate biological conditions. Fewer benchmarking studies use simulated data [3]. Here, we are doing both types of analysis to mitigate the weaknesses of each approach.

We chose several short-read alignment programs and benchmarked their performance based on how well they correctly mapped short (150-nucleotide × 2, 100-nucleotide × 2) to very short (50-nucleotide × 2, 25-nucleotide × 2) NGS-style paired-end reads as well as how they handled a ChIP-seq data set. BWA, Bowtie2, Gsnap, Subread, and Chromap are the aligner programs that were tested [4,5,6,7,8]. We chose these aligners based on three criteria: citations, short read useability, and ease of use. The criteria are further detailed in the Methods section. Each of the aligners we chose indexes the reference sequences and not the reads. Indexing the reference has been the more popular choice for aligners as it uses computational resources more judicially [9]. Bowtie2 and BWA both use a Burrows–Wheeler Transform (BWT) to index the reference. Chromap, Gsnap and Subread use hashing. For aligning, Bowtie2 uses a Needleman–Wunsch algorithm. BWA and Chromap use a Dynamic Programming algorithm. Gsnap uses a Non-DP Heuristic algorithm. Subread uses a Smith–Waterman algorithm [4,5,6,7,8]. While we recognize that a study of how aligners are built algorithmically is important, our aim is to provide information to those who are neophytes and do not necessarily need to understand all the bioinformatical specifics, but for learning about alignment algorithms, we recommend the following review papers [9,10].

The ChIP-seq data set used in our analysis was taken from the ENCODE consortium (see data availability section for accession number) [11]. These data were used to test if depending on the alignment program used, downstream analysis and results would be different.

**Figure 1:**
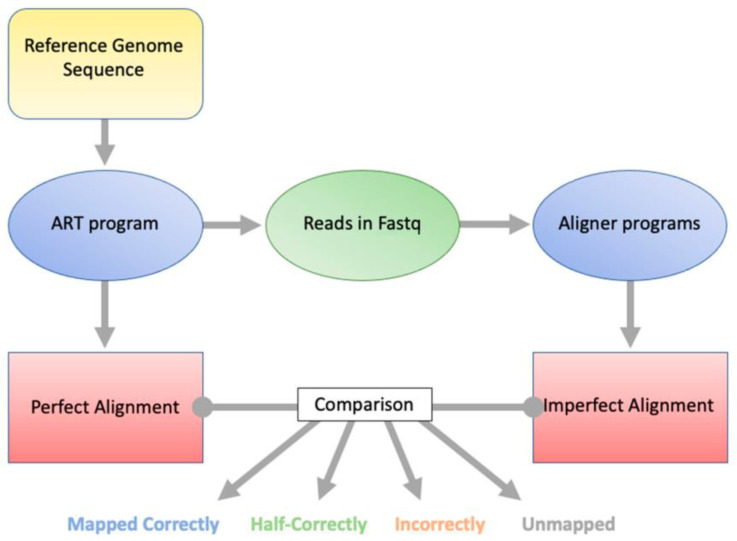
The selected reference genome is used by ART to produce Illumina-style reads in a fastq format. Additionally, a perfect alignment file is produced by ART designating the true genomic loci from which each read was derived. The ART generated fastq file is aligned to the reference genome, and then custom scripts are used to evaluate how well each alignment program did (Imperfect Alignment) compared to the Perfect Alignment file.

The program ART was used to generate NGS-style reads from the *H. sapiens* (human) genome GRCh38p14 [12]. ART takes a reference sequence file and generates reads from it. It also generates a perfectly mapped SAM file for the ART-generated reads, thus allowing the user to know from which loci the reads were generated. The reads can then be run through an alignment program, and the output can be compared to the perfectly mapped SAM file alignment. This allows classification and quantification of how many reads were mapped correctly, mapped half-correctly, mapped incorrectly, or not mapped (Figure 1). We define “mapped correctly” as both paired end reads aligned to the same sequence from which they were generated, “mapped half-correctly” as one of the paired ends aligned to the same sequence from which it was generated, “mapped incorrectly” as aligned to a different sequence than the one from which it was generated, and “unmapped” as neither paired-end read aligned to the reference genome. We will use these definitions henceforth. Bioinformatically, there is no difference between mapped correctly and mapped incorrectly, as the alignment program, in both cases, aligns the reads. However, this distinction is used because there is a difference biologically.

## 2. Results

### 2.1. ChIP-Seq Comparison

Raw ChIP-seq data were aligned with each of the alignment programs. Following the alignment, Peaks were called with MACS2 [13]. Table 1 shows how many peaks were called using each aligner program. Default parameters for each of the aligners were used to align the reads, and the same MACS2 parameters were used to process all five alignments and call peaks.

MACS2 called the fewest peaks from the Gsnap alignment with only 273, and further analysis showed that no peaks were called in chromosomes 9 through 23. The other four alignments resulted in between 74,388 and 76,915 peaks called, which is a small discrepancy of ~3% the in number of peaks. Overall, Bowtie2 and BWA alignments resulted in highly similar peak numbers and positions, with Bowtie2 finding only 340 peaks (0.44%) that were not found by BWA, and BWA finding only 195 peaks (0.26%) that were not found by Bowtie2. A total of 6315 peaks (8.31%) found with the Bowtie2 alignment were not found using the Subread alignment, while the Subread alignment resulted in only 351 peaks that were not found by Bowtie2. The Chromap alignment resulted in slightly fewer peaks than the other previously mentioned alignments but had a similar performance to Subread in that 6020 peaks (8.31%) from the Bowtie2 alignment were not found using the Chromap alignment. Also, the Chromap alignment resulted in 140 peaks that were not found using the Bowtie2 alignment. Finally, the Gsnap alignment data resulted in a tremendous paucity of peaks across the genome (Supplemental Figure S1), and the peaks that were produced using its read output were extremely broad. 

**Figure 2:**
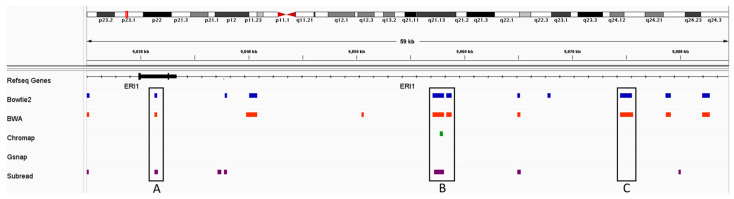
Peaks called at the chr8:9,024,498–9,084,425 locus. At the top of the figure, the entirety of chromosome 8 is represented with a very subtle red box demarking their locus, which is depicted below. Boxes A, B, and C highlight instances of congruent and incongruent peaks called from the alignments generated by the various aligner programs.

As a typical example, Figure 2 shows peaks called by MACS2 using all five read aligners’ read alignments data at the chr8:9,024,498–9,084,425 locus. Peaks called from the Bowtie2 and BWA alignments result in peaks that are usually very similar. As exemplified in Figure 2, box C, both Bowtie2 and BWA alignments result in calling this peak, but using the data from the other three aligners, MACS2 does not find it. While Bowtie2 and BWA alignments often result in the same peaks, a very small minority of the peaks from these alignments are incongruent (<0.5%). Figure 2 box A is an example of where these two alignments result in different peaks being called. Figure 2 box B shows a peak that was called in all alignments except for the alignment generated by Gsnap. In box B, however, we see that the width of the peaks varies between alignments. These data show that even when similar number of peaks are called, the differences in the alignment step due to the use of different aligner programs result in different peaks being called and almost certainly in different biological interpretations, especially when looking at individual loci.

### 2.2. Generated Read Comparison

To directly analyze the accuracy of each genome aligner we used both the top-level assembly of the human genome and the primary assembly to generate in silico reads. The top-level assembly contains non-chromosomal contigs, patches, and haplotypes that represent minor alleles and other variations between human genomes. In the top-level assembly, these extra-chromosomal contigs are not part of the 24 chromosomal contigs/scaffolds representing the 22 autosomes and the X and Y chromosomes, but they are annotated and numbered separately. The primary assembly contains only the assembled chromosomes for the consensus, haploid human genome (24 contigs/scaffolds). ART was used separately on both assemblies to generate sequence reads, and the SAM files generated by ART (Perfect Alignment) were compared to the SAM files generated by the alignment programs using custom python programs available at the GitHub address provided below. We performed this analysis in triplicate. For each read length (150-nucleotide × 2, 100-nucleotide × 2, 50-nucleotide × 2, and 25-nucleotide × 2), three separate libraries were generated with ART, and then each library was aligned individually and compared to the perfect alignment data. The output of these analyses showed which reads the aligners mapped correctly, half-correctly, incorrectly, and did not map (unmapped). 

### 2.3. Accuracy

**Figure 3:**
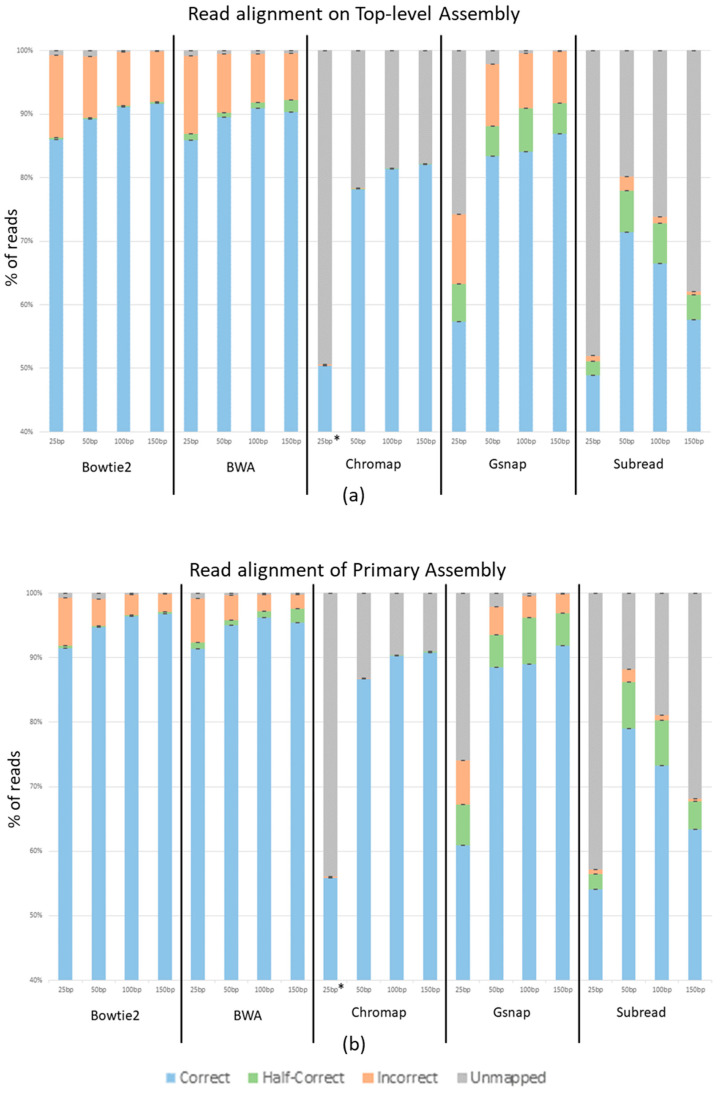
Percent of reads mapped correctly, half-correctly, incorrectly and unmapped. (**a**) shows reads generated from the top-level assembly aligned to the top-level assembly. (**b**) shows reads generated from the primary assembly aligned to the primary assembly. Each graph shows the percentage of reads that mapped correctly (Blue), half-correctly (Green), incorrectly (Orange) and unmapped (Gray). The aligner used and read length are designated on the x-axis. In both (**a**,**b**) the * denotes the use of non-default parameters.

The average percentage of reads across the three replicates that aligned correctly, aligned half-correctly, aligned incorrectly, and were unmapped for each program at each read length is shown in Figure 3. Panel A of Figure 3 shows reads generated from and aligned to the top-level human genome assembly, while panel B shows reads generated from and aligned to the Primary human genome assembly. Bowtie2 and BWA show a 5% increase in correctly mapped reads from the top-level assembly to the primary assembly. Gsnap shows a 4–6% increase depending on the read length. Chromap shows a 9% increase and Subread shows a 6% increase. Other than the overall better performance with the primary assembly, the general trends remain the same between the two genome assemblies.

Unsurprisingly, Figure 3 shows that as the read length increases, so does the percent of correctly mapped reads, while the percent of incorrectly mapped and unmapped reads decreases. Using the Top-level Assembly, Bowtie2 shows 85.9%, 89.2%, 91.2%, and 91.7% of reads correctly mapping at 25 nt, 50 nt, 100 nt, and 150 nt, respectively. It also shows 12.9%, 9.6%, 8.3%, and 7.9% of reads incorrectly mapping at 25 nt, 50 nt, 100 nt, and 150 nt. The BWA output is extremely close to that of Bowtie2 with 85.9%, 89.5%, 90.9%, and 90.3% of reads correctly mapping and 12.2%, 9.2%, 7.6%, and 7.2% incorrectly mapping at 25 nt, 50 nt, 100 nt, and 150 nt, respectively. Chromap shows 50.4%, 78.2%, 81.4%, and 82.0% of reads correctly mapping and 0.2%, 0.05%, 0.002%, and 0.001% of reads incorrectly mapping at 25 nt, 50 nt, 100 nt, and 150 nt, respectively. Gsnap shows 57.3%, 83.4%, 84.1%, and 86.9% of reads correctly mapping and 10.9%, 9.8%, 8.6%, and 8.1% of reads incorrectly mapping at 25 nt, 50 nt, 100 nt and 150 nt, respectively. These trends are seen in all aligners except Subread, which shows a dramatic decrease in unmapped reads as the read length increases; however, Subread, like the other aligners, also shows a substantial decrease in incorrectly mapped reads as the read lengths increase. With the Top-level Assembly, Subread shows 48.9%, 71.4%, 66.5%, and 57.6% of reads correctly and 0.9%, 2.1%, 0.9%, and 0.5% of reads incorrectly mapping at 25 nt, 50 nt, 100 nt, and 150 nt. While Bowtie2 and BWA performed similarly, we see that the amount of half-correct reads was greater in the BWA data set. In the Top-level Assembly, Bowtie2 shows half-correct-mapping reads decreasing from 3% at 25 nt to 0.2% at 150 nt of the reads, while BWA shows 9% decreasing to 1.9% of the reads being half-correct as the read lengths increase from 25 to 150 nt. It is noteworthy that the increase in half-correct reads in BWA is approximately the same percent as the difference between the incorrectly mapping reads between Bowtie2 and BWA. Finally, Chromap had very few incorrectly mapping reads, from 0.2% for the 25 nt read length to 0.001% for the 150 nt read length, using the Top-level Assembly.

### 2.4. Further Analysis

**Figure 4:**
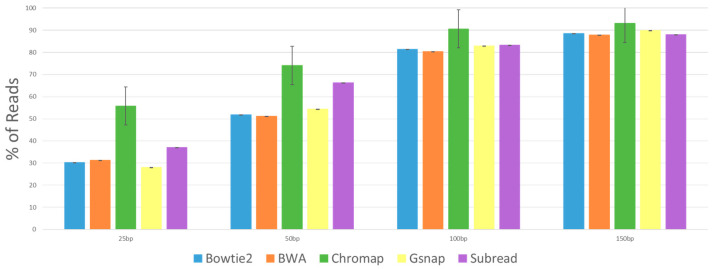
Percent of incorrectly mapped reads with errors in the sequence. Blue is Bowtie2, Orange is BWA, Green is Chromap, Yellow is Gsnap and Purple is Subread. On the x-axis, the 25 nt read length results are the first set, which are followed by the 50 nt, 100 nt and 150 nt read lengths data.

We suspected and wanted to test if the incorrectly mapped reads were caused by errors in the sequencing. ART builds in errors at a rate similar to known Illumina error rates and records these “sequencing errors” in the Perfect Alignment SAM file in the CIGAR score column. Thus, using custom scripts, we determined which of the sequence reads that did not align correctly (the incorrectly mapped reads) had “sequencing errors”. Figure 4 shows the percentage of the incorrectly mapped reads that have errors in the sequences, and we see that all the aligner programs reported similar rates. Chromap shows higher rates of incorrectly mapped reads that have errors. As might be expected, there is a trend that all aligners show that with longer reads come higher rates of incorrectly mapped reads that have errors, and that at the 150 bp read length, about 90% of the incorrectly mapped reads had “sequence errors,” confirming that as the read length increases, the propensity for error-free reads to align incorrectly drops dramatically.

A genomic analysis of incorrectly mapped reads did not provide conclusive evidence for or against specific genomic elements that were more likely to have these incorrectly mapped reads mapped to them. When viewed at the whole genome level, incorrectly mapped reads do pile up at distinct genomic loci but do not result in localized peaks when analyzed with MACS2 (data not shown). These read piles are well conserved between Bowtie2 and BWA, which is unsurprising because of the similarities in the ways these programs map reads (Supplemental Figure S2). When these piles are compared to all the ART-generated reads (not mismatched but mapped to their loci of origin), we observe that these piles seem to be frequently flanking areas of the genome that were masked both in the generation of reads and in the mapping of reads (Supplemental Figure S2). When looking at incorrectly mapped reads and a base pair resolution, there is little to no difference between the ART reads and the aligner mapped reads (Supplemental Figure S3). Thus, it is hard to conclude that incorrectly mapped reads are due to some genomic elements.

### 2.5. Accessibility

Finally, as part of the purpose of this study was to see which of our tested aligners would be easiest for new informaticians to work with, we assessed the ease of use based on the following: (1) the quality of the documentation provided by the program, (2) if the program is still being supported, and (3) if the program was able to work with little to no help. We realize that this is anecdotal data and would vary based on the experience of the user. BWA, Bowtie2 and Chromap were all very well-documented, very well supported, and extremely easy to use. The rest of the aligners were well-documented, somewhat supported, and moderately easy to use. In general, based on ease of use, we would recommend BWA, Bowtie2 and Chromap for novice users, and we would recommend the other aligners to those that have some experience with computational biology.

## 3. Discussion

Overall, the aligners did well in correctly mapping short reads. With longer read lengths, the aligners, generally, mapped more reads correctly. While this is expected, the increase in accuracy is quite dramatic: from 85.9% to 91.7% in Bowtie2 and similar in the other aligners, except Subread. When possible, we recommend using paired end reads of at least 100 nt, and we observe that the gains associated with 150 nt reads are minimal. Our ART-generated reads were derived from DNA with a 251 bp fragment size. This size was used because it more closely follows typical fragment lengths found in ChIP-seq data sets. We do recognize that ATAC-seq data sets consist of mostly shorter DNA fragments, and read lengths of 100 nt may not yield any better accuracy for those shorter fragments but could substantially increase the accuracy of the alignment for reads from longer fragments. 

We see an increase in accuracy in aligned reads with the reads generated from the Primary genome assembly when compared to those generated from the Top-level genome assembly. This is likely due to the primary assembly not having confounding contigs to which reads can be mismatched. The top-level assembly contains non-chromosomal contigs, patches, and haplotypes that represent minor alleles and other variations between human genomes, whereas the primary assembly contains only the assembled chromosomes for the consensus human genome. Thus, in some instances, reads with “sequence errors” derived from one chromosome or non-chromosomal contig in the top-level assembly could map better to the orthologous sequence (the contig or chromosome sequences, respectively) and be classified as mapped incorrectly. For ATAC-seq, ChIP-seq and MNase-seq-type experiments, aligning to the top-level assembly could therefore result in two peaks called when in biological terms, there is only one. We recommend using the primary assembly for these types of experiments to avoid this issue. 

When looking at the half-correct alignments, we see varied results. Bowtie2 and Chromap have low numbers of half-correct reads, around 0.3% and 0.1%, respectively. BWA is slightly higher at 1%, and Gsnap and Subread have significant half-correct subsets, each at around 5%. As previously stated, each of these programs was run with default parameters, and each program handles fragment sizes differently. This variable is possibly the cause of the large half-correct subsets in BWA, Gsnap and Subread. We strongly encourage understanding aligners’ parameters so that the best results can be achieved for specific experimental conditions. We also suggest using our provided custom python programs and ART to mimic specific data sets and experiment with parameters that could result in better accuracy for each experiment. We did not adjust parameters in this study because default parameters are set as default by the program designers because they are expected to be the most likely used and thus most useful in general circumstances. Also, given the number of potential parameter combinations, it was not practical for us to even begin to test them.

Chromap produced incredibly low amounts of incorrect reads. For example, in the 150 nt data set, Bowtie2, BWA and Gsnap had hundreds of thousands of incorrect reads. Subread had tens of thousands of incorrect reads, and Chromap had hundreds of incorrect reads. While there is a substantial increase in unmapped reads, the overall accuracy of Chromap makes it a compelling program to use. The large number of unmapped reads (and potentially missed biological data) from Chromap most likely can be compensated for by deeper sequencing unless Chromap shows some bias in the reads it does not map (which we did not detect). It should also be noted that Chromap did not allow for reads below 36 nt without altering the default parameters (denoted by * in Figure 3). This could explain why only ~50% of the 25 nt reads mapped correctly.

In our study, the telomeric and repeat regions of the human reference genome used to generate the ART reads were annotated as non-specific nucleotides (N); in other words, a hard masked reference genome was used. For this reason, ART excluded these regions in the generation of the NGS libraries. We recommend that reads mapped to regions identified in the ENCODE blacklist should not be used in downstream analysis in ATAC-seq, ChIP-seq, or MNase-seq experiments [14].

The high rates of reads with errors in the incorrectly mapped read sets indicate that the aligner program is incorrectly mapping reads because of the sequencing errors. There are two ways in which the potential problems from these incorrectly mapped reads could be overcome: first, with better sequencing quality, and second, with increased sequencing depth. More reads would not change the rate of errors, but if sequencing errors are near-random events, it would dilute the effect that the incorrectly mapped reads have on downstream analysis. When we look at the incorrectly mapped reads with errors (Figure 4), we see that Chromap has a slightly higher rate than the other aligners and that the rates go up as the read length increases. The number of incorrectly mapped reads generated from Chromap was extremely small compared to the other aligners, while the number of reads not mapping was proportionally much higher. This likely indicates that Chromap has very stringent read quality control and will not even attempt to map reads that are suspect, due to their quality scores, of having sequencing errors. But when reads are incorrectly mapped by Chromap, it is usually (over 90%) due to sequence errors. Since the numbers of incorrectly mapped reads with Chromap at 100 nt and 150 nt lengths are so incredibly low (0.002% and 0.001%), these incorrectly mapped reads are of little biological concern, especially if complemented with increased sequencing depth.

Finally, we were not able to conclusively determine if incorrectly mapped reads mapped to particular genomic elements or loci. These inclusive results are likely due to the relatively low depth of coverage used in our simulated read analysis. Whereas it is possible to do much deeper coverage analysis on biological data from MNase-seq, ChIP-seq or ATAC-seq data (due to the vast majority of reads mapping to only a small subset of the genome) and thus see peaks, a deep-coverage analysis is not practical with simulated ART reads, as ART generates reads across the entire non-masked portion of the genome and not a small subset of it. Such an analysis will most likely not be possible until a read generator is developed to specifically simulate short-read data sets (i.e., MNase-seq, ChIP-seq or ATAC-seq data sets).

## 4. Materials and Methods

The five alignment programs used for comparison were chosen based on several criteria. We included only aligners that specialize in short reads, as this study is concerned with finding the best program for ChIP-seq, ATAC-seq, and MNase-seq experiments. Aligners that required read lengths longer than 50 nt were omitted. Furthermore, aligners that are not equipped to handle indels and substitutions were not included, as those are the most common sequencing errors with Illumina sequencing [15]. 

In addition to the criteria listed above, aligner programs were also vetted based on popularity and ease of use. With the exception of Chromap, we chose commonly (meaning the aligner has been cited at least 100 times) used aligners. Chromap was originally published in 2021 and was included because it was designed specifically for ATAC-seq and ChIP-seq data. Also, aligners whose documentation was no longer available or was deemed insufficient were omitted. Furthermore, each aligner was used with default parameters, or close to default, when user-designated parameters were required. A complete set of aligner usage is found in the supplemental section (Supplemental File S1).

Raw ChIP-seq data were aligned with each of the individual alignment programs. Following the alignment step, SAM files were converted to BAM with Samtools [16], and duplicate reads were removed with Picard MarkDuplicates [17]. Peaks were called with MACS2 with the default parameters except: no control, paired end bam, and nomodel. BED files were visualized using the IGV genome browser [18,19,20,21]. The number of peaks is reported in Table 1. To calculate the number of unique peaks generated from each alignment, peaks were called again with MACS2 with the same parameters as above but this time with the other alignments as a control (Table 2). This shows the differential peak number between any two alignments or more specifically the number of peaks that are in the test alignment that are not in the control alignment.

We used the program ART to generate Illumina-type sequence reads. Illumina-type reads were chosen because of the prevalence of Illumina sequencing currently in the chromatin field. We generated reads with an average fragment length of 251 bp with a 10 bp standard deviation. This length was used to mimic the length of fragments found in ChIP-seq experiments. Furthermore, for the same reason, read lengths of 25, 50, 100 and 150 nt were used, and all reads were paired end.

When generating reads, ART incorporates errors, both substitutions and indels, into the reads. The amount of substitution errors can be set manually or by built-in algorithms that follow typical read error rates for different platforms. For this study, we used error rates that follow the Illumina HiSeq 2500 platform. These substation error rates were increased by one-tenth to ensure that there were enough errors to analyze without making the data unrealistic. The number of indels was set to follow typical Illumina indel error rates.

The number of reads generated was set to adequately cover the human genome while keeping the computational workload within working limits. We chose the *Homo sapiens* genome because of the large amount of available data generated from ATAC, MNase, and ChIP-seq projects completed with human samples and because of the complexity of the human genome. For the analysis, the desired fold-coverage was used to generate the number of reads instead of total read count to allow for even coverage across the entire genome for the various read lengths tested. When generating ART reads by total read count, each chromosome in the reference file will have the same total number of reads regardless of chromosome size. This would have been a problem with the human genome because chromosome size varies greatly. A complete set of ART usage and parameters can be found in the supplemental section (Supplemental File S1).

ART generates a SAM file with the generated reads perfectly aligned to their genomic loci of origin. We compared the SAM file generated by ART to the SAM files generated by the alignment programs using custom-made Python programs. These programs look at the locus of the read from the aligner SAM file and determine if it is the same locus as the ART SAM file. The output of these programs classified the reads the aligners mapped as correctly mapped, half-correctly mapped, and incorrectly mapped. The unmapped classification was taken from the FLAG scores in the aligner SAM files. In the case of Chromap, the SAM file did not report unmapped reads. This is because the SAM output is not the default for Chromap, BED is, and there is no way to report unmapped reads in a BED file. Therefore, unmapped reads for Chromap were calculated by subtracting the sum of incorrect, correct, and half-correct reads from the total number of reads in the fastq files. The custom-made Python programs are available at the provided GitHub address.

In order to analyze where incorrectly mapped reads were mapping to the genome, ART-generated SAM files (perfect alignment reads) and aligner program-generated SAM files of incorrectly mapped reads were converted to BigWig files with DeepTools version 3.5.2 [22] and viewed with the IGV genome browser.

## 5. Conclusions

We recommend the use of Bowtie2, BWA and Chromap, as they have all performed well under default parameters, even to the point that a novice should feel comfortable using them. However, as with all science, it is still necessary to do due diligence and not take these programs for granted. We recommend first understanding the parameters of the programs and then testing them to see what settings work best for specific data sets using ART to simulate reads. Furthermore, when at all possible, read lengths of 100 nt or larger should be used.

Here, we have provided a methodological overview and custom Python scripts that should enable a neophyte to use ART to produce simulated reads that match the characteristics of their biological data, use the tested aligner programs to map the reads, and then use our custom Python scripts to evaluate the effectiveness of each aligner. By doing so, one can make a data-based decision on which aligner program to use to map their biological reads.

## Figures and Tables

**Table 1:** Number of peaks from each alignment.

Aligner	Number of Peaks
Bowtie2	76,915
BWA	76,136
Chromap	74,388
Gsnap	273
Subread	76,007

**Table 2:** Number of unique peaks from each alignment.

Aligner: Control	Number of Unique Peaks
Bowtie2: BWA	340
BWA: Bowtie2	195
Bowtie2: Subread	6315
Subread: Bowtie2	351
Bowtie2: Chromap	6020
Chromap: Bowtie2	140

## Data Availability

The data sets used and/or analyzed in the study are available from the corresponding author upon request. ENCODE Data Coordination Center (DCC) accession number doi:10.17989/ENCSR835MMN Available online: https://github.com/Johnson-Lab-BYU/Aligner_Benchmarking (accessed on 4 September 2023).

## Supplementary Materials

The following supporting information can be downloaded at: https://www.mdpi.com/article/10.3390/ijms241814074/s1.
