# Supplementary material for: Short Sequence Aligner Benchmarking for Chromatin Research
Source: Int J Mol Sci. 2023 Sep 14;24(18):14074. doi: 10.3390/ijms241814074 (PMC10531285; doi:10.3390/ijms241814074)
Supplement: Supplementary file 1 [file ijms-24-14074-s001.zip › ijms-2587019-supplementary.pdf]

Supplementary File S1

Outline and example of how to use this pipeline.

1. Choose genome.
    a. *Home sapiens* GRCh38.p14 build.
2. Run ART on chosen genome.
    a. ./art_illumina -sam -i ~/hs_GRCh38.p14.fa -p -l 25 -f 1 -m 147 -s 10 -o Art_hs_GRCh38_25 -ir 0.0009 -ir2 0.0015 -dr 0.0011 -dr2 0.0023 -na -sp -ss HS25 -qs -10 -qs2 -10
        i. ./art_illumina – command for art illumina simulation
        ii. -sam – command to include sam output
        iii. -I ~/hs_GRCh38.p14.fa - genome fasta file location
        iv. -p – command for paired end reads
        v. -l 25 – command for 25 bp length reads
        vi. -f 1 – command for fold coverage of 1
        vii. -m 147 – command for mean fragments size of 147 bp
        viii. -s 10 – command for 10 bp standard deviation of fragments size
        ix. -o Art_hs_GRCh38_25 – command for output file name
        x. -ir 0.0009 – command for the first-read insertion rate
        xi. -ir2 0.0011 – command for the second-read insertion rate
        xii. -dr 0.0011 – command for the first-read deletion rate
        xiii. -dr2 0.0023 – command for the second-read deletion rate
        xiv. -na – command for not outputting an ALN alignment file
        xv. -sp – command to indicate use of separate quality profiles for different bases
        xvi. -ss HS25 – command to use the built-in Illumina sequencing system HS25 profile
        xvii. -qs -10 – command to shift the first-read quality score by -10
        xviii. -qs2 -10 – command to shift the second-read quality score by -10
3. Run aligner on ART produced fastq files.
    a. Index genome
        i. ./bowtie2-build --large-index --threads 8 ~/hs_GRCh38.p14.fa ~/bowtie2/hs_GRCh38
            1. ./bowtie2-build – command to build bowtie2 index
            2. --large-index – command to use the large-index modification
            3. --threads 8 – command to use 8 processing threads
            4. ~/hs_GRCh38.p14.fa – command to find genome location
            5. ~/bowtie2/hs_GRCh38 – command to name output
    b. Map reads
        i. ./bowtie2 -p 8 -x ~/bowtie2/hs_GRCh38/hs_GRCh38 -1 ~/Art_hs_GRCh38_25_1.fastq -2 ~/Art_hs_GRCh38_25_2.fastq -S Bowtie2_hs_25.sam
            1. ./bowtie2 – command to run bowtie2 alignment
            2. -p 8 – command to use 8 processing threads
            3. -x ~/bowtie2/hs_GRCh38/hs_GRCh38 – command to locate the index
            4. -1 ~/Art_hs_GRCh38_25_1.fastq – command to locate the first read fastq file

        5. -2 ~/Art_hs_GRCh38_25_2.fastq – command to locate the second read fastq file
        6. -S Bowtie2_hs_25.sam – command to name the output file
4. Sort ART SAM and aligner SAM with Linux sort command.
    a. sort -o Art_hs_GRCh38_25_p1_sort.sam Art_hs_GRCh38_25_p1.sam
        i. sort – Linux command to sort
        ii. -o Art_hs_GRCh38_25_p1_sort.sam – command for output file name
        iii. Art_hs_GRCh38_25_p1.sam – command for input file name
    b. Repeat with Bowtie2 sam file
5. Run SamCompare4.6.py on ART produced SAM and aligner Produced SAM
    a. Python Art_hs_GRCh38_25_p1_sort.sam Bowtie2_hs_25_sort.sam ~/Bowtie2_output ~/Bowtie2_output/hs_25
        i. Python – command to run python.
        ii. Art_hs_GRCh38_25_p1_sort.sam – location of the art generated sam file
        iii. Bowtie2_hs_25_sort.sam – location of the aligned sam file
        iv. ~/Bowtie2_output – name of directory where you want the output to go.
        v. ~/Bowtie2_output/hs_25 – name of the output file

Usage

ART: https://www.niehs.nih.gov/research/resources/software/biostatistics/art/index.cfm

./art_illumina -sam -i ~/hs_GRCh38.p14.fa -p -l 25 -f 1 -m 147 -s 10 -o Art_hs_GRCh38_25 -ir 0.0009 -ir2 0.0015 -dr 0.0011 -dr2 0.0023 -na -sp -ss HS25 -qs -10 -qs2 -10

./art_illumina -sam -i ~/hs_GRCh38.p14.fa -p -l 50 -f 1 -m 147 -s 10 -o Art_hs_GRCh38_36 -ir 0.0009 -ir2 0.0015 -dr 0.0011 -dr2 0.0023 -na -sp -ss HS25 -qs -10 -qs2 -10

./art_illumina -sam -i ~/hs_GRCh38.p14.fa -p -l 100 -f 1 -m 147 -s 10 -o Art_hs_GRCh38_50 -ir 0.0009 -ir2 0.0015 -dr 0.0011 -dr2 0.0023 -na -sp -ss HS25 -qs -10 -qs2 -10

./art_illumina -sam -i ~/hs_GRCh38.p14.fa -p -l 150 -f 1 -m 147 -s 10 -o Art_hs_GRCh38_50 -ir 0.0009 -ir2 0.0015 -dr 0.0011 -dr2 0.0023 -na -sp -ss HS25 -qs -10 -qs2 -10

BWA: https://bio-bwa.sourceforge.net/

Indexing:

./bwa index -a bwtsw <*Reference file*>

Aln file generation:

./bwa aln <*Path to Reference Directory*> <*Read file in FASTQ format*> > <*output.sai*>

Mapping:

./bwa sampe <*Path to index Directory*> <*Path to first .sai file*> <*Path to second .sai file*> <*Path to first .fq file*> <*Path to second .fq file*> > <*output.sam*>

Bowtie2: https://bowtie-bio.sourceforge.net/bowtie2/index.shtml

Indexing for small genomes:

./bowtie2-build *<path to Reference file>* *<output>*

Indexing for large genomes:

./bowtie2-build --large-index --threads 8 *<path to Reference file>* *<output>*

Mapping:

./bowtie2 -p 8 -x *<Path to index Directory>* -1 *<Path to first .fq file>* -2 *<Path to second .fq file>* -S *<output.sam>*

Gsnap: https://bioinformaticshome.com/tools/rna-seq/descriptions/GSNAP.html#gsc.tab=0

Indexing:

./gmap_build -d *<output>* *<path to Reference file>*

Mapping:

./gsnap -A sam -D *<Path to index Directory>* -d *<index prefix>* *<Path to first .fq file>* *<Path to second .fq file>* > *<output.sam>*

Subread: https://subread.sourceforge.net/

Indexing:

./subread-buildindex -o *<output>* *<path to Reference file>*

Mapping:

./subread-align -t 1 -d 50 -D 600 -i *<Path to index>* -r *<Path to first .fq file>* -R *<Path to second .fq file>* -o *<output.bam>*

Chromap: https://github.com/haowenz/chromap

Indexing:

./chromap -i -r *<path to Reference file>* -o hs.index

Mapping:

./chromap --preset chip -x *<Path to index>* -r *<path to Reference file>*-1 *<Path to first .fq file>* -2 *<Path to second .fq file>* --SAM -o *<output.sam>*

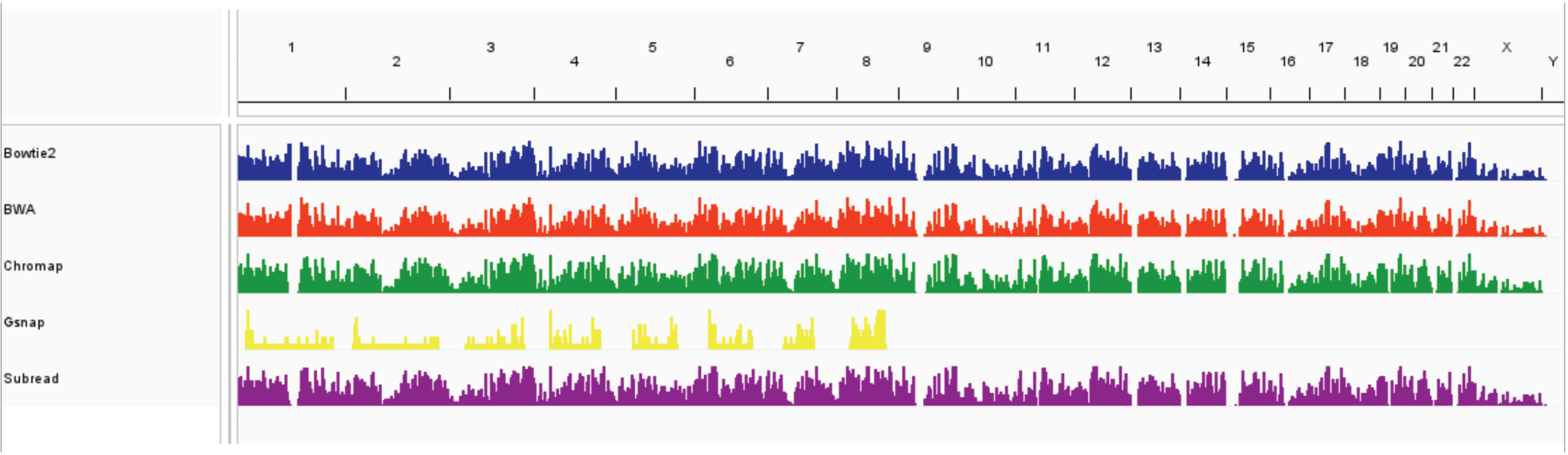

Supplemental Figure S1. ChIP-seq peaks across the genome; Bowtie2 is shown in blue, BWA in orange, Chromap in green, Gsnap in yellow, and Subread in purple. The x-axis is the human genome with each chromosome marked. The y-axis shows the peak amplitude.

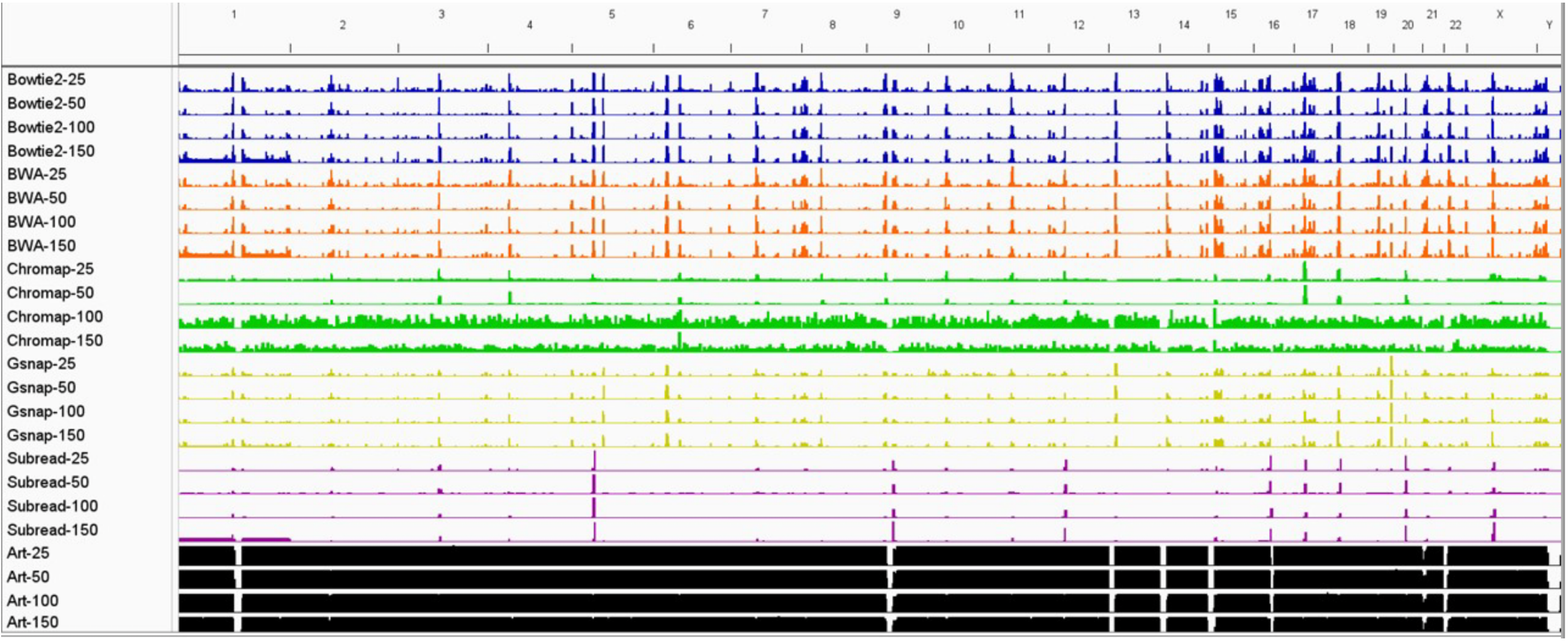

Supplemental Figure S2. Incorrect reads BigWig and Art file BigWig across the genome; Bowtie2 is shown in blue, BWA in orange, Chromap in green, Gsnap in yellow, Subread in purple, and ART in black. For each aligner and ART 25nt, 50nt, 100nt, and 150nt read length data sets. The x-axis is the human genome with each chromosome marked. The y-axis shows the coverage of incorrect reads for the aligners, and coverage of the total data for the ART set.

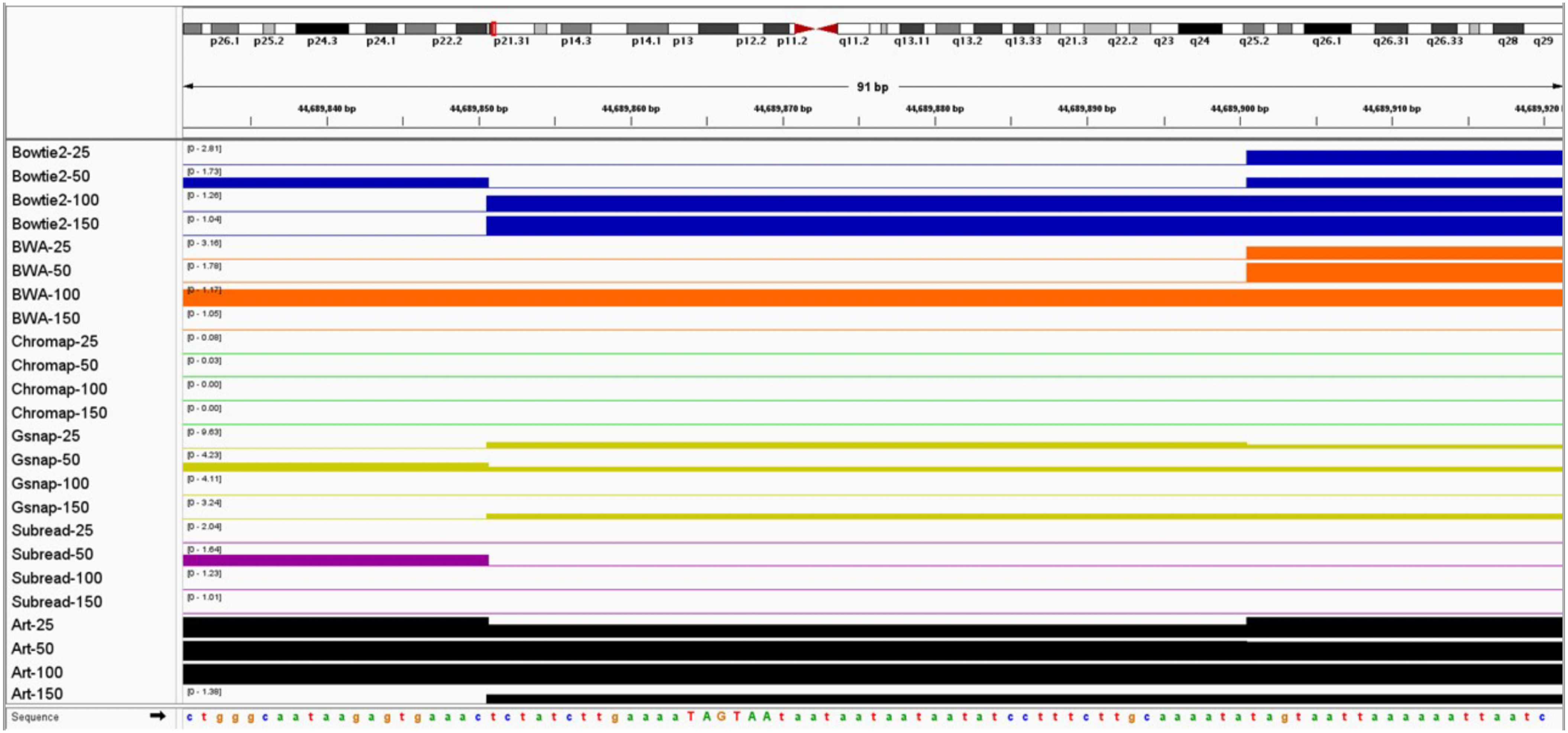

Supplemental Figure S3. Incorrect reads BigWig and Art file BigWig examples at base pair resolution; Bowtie2 is shown in blue, BWA in orange, Chromap in green, Gsnap in yellow, Subread in purple, and ART in black. For each aligner and ART 25nt, 50nt, 100nt, and 150nt read length data sets are shown. The x-axis is the human chromosome 3 at 44,689,830-44,689,921 loci. The y-axis shows the coverage of incorrect reads for the aligners, and coverage of the total data for the ART set.
